# Morphine‐induced cutaneous adverse drug reaction following occupational diacetylmorphine contact dermatitis: A case report

**DOI:** 10.1111/cod.13302

**Published:** 2019-05-24

**Authors:** Ewoud van den Hoed, Pieter Jan Coenraads, Marie L. A. Schuttelaar

**Affiliations:** ^1^ Department of Dermatology University of Groningen, University Medical Centre Groningen Groningen The Netherlands

**Keywords:** case report, cross‐reaction, cutaneous adverse drug reaction, diacetylmorphine, drug eruption, heroin, morphine, opiates, systemic allergic dermatitis

Contact dermatitis caused by diacetylmorphine (heroin) and by morphine have both been described before.^1^, ^2^, ^3^, ^4^, ^5^ In addition, a case of acute generalized exanthematous pustulosis (AGEP) as a reaction to morphine has previously been described.^6^ The present report describes a patient who was occupationally sensitized to diacetylmorphine, and developed a skin reaction after receiving morphine following surgery.

## CASE REPORT

A 55‐year‐old woman with a history of occupational contact dermatitis caused by diacetylmorphine underwent surgery for a mammary carcinoma. In the past, she had worked at a municipal treatment centre for drug addicts until 2000. At that time, she developed allergic contact dermatitis after contact with diacetylmorphine. Patch testing was performed with our departmental extended European baseline series (TRUE Test panels 1 and 2, supplemented with additional investigator‐loaded allergens), a cosmetic series, and an opiate series (SmartPractice Europe, Reinbek, Germany). All investigator‐loaded allergens were tested in Van der Bend square chambers (Van der Bend, Brielle, The Netherlands), and all patch tests were attached to the back with Fixomull stretch (BSN Medical, Hamburg, Germany) for 2 days. Readings were performed on day (D) 2 and D3.

Patch testing showed positive reactions to morphine hydrochloride, morphine, diacetylmorphine, and codeine dihydrophosphate (Table 1). The patient was one of the occupation‐related cases described previously.^1^, ^2^ Sixteen years later, she underwent a mastectomy. One day after surgery, she developed an erythematous papular itchy facial rash, which spread over the body on the next day. In addition, vesicles were seen on the face and neck. No signs of fever, laryngitis or pustules were seen. The patient was treated with clobetasol ointment for 1 week, with good results.

**Table 1:** Patch test results with the opiate series obtained in 2000

Allergen	Concentration (%)	Vehicle	Day 2	Day 3
Fentanyl citrate	0.1	pet.	−	−
Oxycodone	1.0	pet.	−	−
Piritramide (ampoule, 10 mg/mL aq.)	“as is”		−	−
Buprenorphine	0.1	pet.	−	−
Pentazocine (ampoule, 30 mg/mL aq.)	“as is”		−	−
Methadone	1.0	pet.	−	−
Tramadol	1.0	pet.	−	−
Caffeine	0.5	pet.	−	−
Morphine hydrochloride	1.0	aq.	?+	+
Morphine (10 mg) powder	“as is”		?+	+
Diacetylmorphine	1.0	pet.	?+	+
Codeine dihydrophosphate	1.0	eth.	?+	+

Abbreviation: eth., ethanol 70%.

During mastectomy, the patient had received morphine, cefazolin, dexamethasone, naproxen, propofol, paracetamol, remifentanil, and rocuronium. After surgery, she was treated with naproxen, paracetamol, pantoprazole, fraxiparine, and ondansetron. She had never reacted to paracetamol, ibuprofen, or naproxen. According to Litt's drug eruption & reaction database, delayed‐type reactions to and exanthemas caused by the other administered drugs are very rare.^7^ Hypersensitivity to propofol is also rare, and is almost always reported in terms of anaphylactoid reactions or anaphylaxis^8^ We assume that the patient developed a morphine‐induced delayed‐type cutaneous adverse drug reaction following occupational diacetylmorphine contact dermatitis. An intravenous provocation test to reproduce the skin reaction was not performed, because of the patient's severe rash.

## DISCUSSION

We assume that the patient described here developed delayed‐type (type IV) systemic allergic dermatitis caused by morphine in the context of her past occupational contact sensitization to diacetylmorphine. In 2000, besides stopping occupational exposure, the patient was advised to avoid treatment with morphine, diacetylmorphine, and codeine, because of the chemical similarities between these opiates. Nevertheless, she received morphine during surgery in 2016.

Contact dermatitis and AGEP caused by diacetylmorphine and morphine have been reported before.^1^, ^2^, ^3^, ^4^, ^5^, ^6^ Furthermore, the chemical similarities between morphine, diacetylmorphine and codeine are well known.^9^ Thyssen and Maibach, along with Schnyder and Pichler, reviewed the possible pathomechanisms of systemic allergic dermatitis.^10^, ^11^ The exact mechanism remains unclear. However, the pharmacological interactions of drugs with immune receptors concept may explain the predominant skin involvement in T cell‐mediated reactions to systemically applied drugs.^11^

In conclusion, although definite proof by means of a provocation test could not be provided, we feel that reporting this case is of importance to alert those who are occupationally sensitized to opiates to the risk of skin and systemic reactions in cases of future medication with these drugs.

## CONFLICTS OF INTEREST

The authors have no conflicts of interest to report.

## References

[1] Coenraads PJ , Hogen Esch AJ , Prevoo RL . Occupational contact dermatitis from diacetylmorphine (heroin). Contact Dermatitis. 2001;45:114. 11553127 10.1034/j.1600-0536.2001.045002114.x

[2] Hogen Esch AJ , van der Heide S , van den Brink W , van Ree JM , Bruynzeel DP , Coenraads PJ . Contact allergy and respiratory/mucosal complaints from heroin (diacetylmorphine). Contact Dermatitis. 2006;54:42‐49. 16426293 10.1111/j.0105-1873.2006.00745.x

[3] Colomb S , Bourrain JL , Bonardel N , Chiriac A , Demoly P . Occupational opiate contact dermatitis. Contact Dermatitis. 2017;76:240‐241. 28317188 10.1111/cod.12666

[4] Hvid L , Svendsen MT , Andersen KE . Occupational allergic contact dermatitis caused by heroin (diacetylmorphine) and morphine. Contact Dermatitis. 2016;74:301‐302. 27040874 10.1111/cod.12515

[5] Sasseville D , Blouin M , Beauchamp C . Occupational allergic contact dermatitis caused by morphine. Contact Dermatitis. 2011;64:166‐168. 21272024 10.1111/j.1600-0536.2010.01827.x

[6] Kardaun SH , de Monchy JG . Acute generalized exanthematous pustulosis caused by morphine, confirmed by positive patch test and lymphocyte transformation test. J Am Acad Dermatol. 2006;55:S23. 10.1016/j.jaad.2006.02.032 16843118

[7] http://www.drugeruptiondata.com. Accessed April 26, 2019.

[8] Asserhøj LL , Mosbech H , Krøigaard M , Garvey LH . No evidence for contraindications to the use of propofol in adults allergic to egg, soy or peanut. Br J Anaesth. 2016;116:77‐82. 26675952 10.1093/bja/aev360

[9] de Cuyper C , Goeteyn M . Systemic contact dermatitis from subcutaneous hydromorphone. Contact Dermatitis. 1992;27:220‐223. 1280549 10.1111/j.1600-0536.1992.tb03249.x

[10] Thyssen JP , Maibach HI . Drug‐elicited systemic allergic (contact) dermatitis—update and possible pathomechanisms. Contact Dermatitis. 2008;59:195‐202. Book. 18844694 10.1111/j.1600-0536.2008.01367.x

[11] Schnyder B , Pichler WJ . Mechanisms of drug‐induced allergy. Mayo Clin Proc. 2009;84:268‐272. 19252115 10.4065/84.3.268 PMC2664605

